# Pediatric Rotavirus A Infection Among 337,019 Participants Within Asia: A Pan‐Asian Systematic Review and Meta‐Analysis

**DOI:** 10.1155/jotm/6627946

**Published:** 2026-04-29

**Authors:** Ali A. Rabaan, Bello E. Kizito, Abdulsalam Alawfi, Amer Alshengeti, Amal H. Alfaraj, Wadha A. Alfouzan, Haya Altawalah, Dalal Meshal Bashah, Alya Aali Almatrafi, Amal Abdulqadir Mujahed, Bandar Alwan Albaradi, Mohammed Garout, Heba A. Alsaffar, Mona A. Al-Zaher, Noor M. Al Sheef, Esraa Z. Al-Nass, Leia Kamal Mohammed AlKhathlan, Hayam A. Alrasheed, Nawal A. AlKaabi, Zainab H. Almansour, Huseyin Tombuloglu

**Affiliations:** ^1^ Molecular and Agnostic Laboratory, Johns Hopkins Aramco Healthcare, Dhahran, 31311, Saudi Arabia, jhah.com; ^2^ College of Medicine, Alfaisal University, Riyadh, 11533, Saudi Arabia, alfaisal.edu; ^3^ Department of Public Health and Nutrition, The University of Haripur, Haripur, 22610, Pakistan, uoh.edu.pk; ^4^ Department of Microbiology, Faculty of Natural Sciences, Kogi State (Prince Abubakar Audu) University, Anyigba, 1008, Nigeria; ^5^ Department of Pediatrics, College of Medicine, Taibah University, Al-Madinah, 41491, Saudi Arabia, taibahu.edu.sa; ^6^ Department of Infection Prevention and Control, Prince Mohammad Bin Abdulaziz Hospital, National Guard Health Affairs, Al-Madinah, 41491, Saudi Arabia, ngha.med.sa; ^7^ Pediatric Department, Abqaiq General Hospital, First Eastern Health Cluster, Abqaiq, 33261, Saudi Arabia; ^8^ Department of Microbiology, Faculty of Medicine, Kuwait University, Safat, 13110, Kuwait, kuniv.edu; ^9^ Microbiology Unit, Department of Laboratories, Farwaniya Hospital, Farwaniya, 85000, Kuwait; ^10^ Clinical Virology Unit, Yacoub Behbahani Centre, Sabah Hospital, Ministry of Health, Kuwait City, 70653, Kuwait, sante.gov.ma; ^11^ Clinical Virology Unit, Department of Microbiology, Faculty of Medicine, Kuwait University, Safat, 13110, Kuwait, kuniv.edu; ^12^ Infectious Diseases Section, Internal Medicine Department, King Abdulaziz Hospital, Jeddah, 22421, Saudi Arabia, ngha.med.sa; ^13^ Laboratory and Blood Bank Department, Maternity and Children Hospital, Makkah, 21955, Saudi Arabia, moh.gov.sa; ^14^ Department of Pediatric Infectious Diseases, King Fahad Specialist Hospital, Dammam, 32222, Saudi Arabia, kfsh.med.sa; ^15^ Department of Community Medicine and Health Care for Pilgrims, Faculty of Medicine, Umm Al-Qura University, Makkah, 21955, Saudi Arabia, uqu.edu.sa; ^16^ Department of Azizia Primary Health Care, Ministry of Health, Dammam, 32211, Saudi Arabia, moh.gov.sa; ^17^ Department of Commitment Management, Directorate of Health Affairs in the Eastern Province, Dammam, 31176, Saudi Arabia; ^18^ International Programs School, Ministry of Health, Dammam, 34236, Saudi Arabia, moh.gov.sa; ^19^ Department of Pharmacy Practice, College of Pharmacy, Princess Nourah Bint Abdulrahman University, Riyadh, 11671, Saudi Arabia, pnu.edu.sa; ^20^ College of Medicine and Health Science, Khalifa University, Abu Dhabi, 127788, UAE, kustar.ac.ae; ^21^ Sheikh Khalifa Medical City, Abu Dhabi Health Services Company (SEHA), Abu Dhabi, 51900, UAE, skmc.ae; ^22^ Biological Science Department, College of Science, King Faisal University, Hofuf, 3198, Saudi Arabia, kfu.edu.sa; ^23^ Department of Genetics Research, Institute for Research and Medical Consultations (IRMC), Imam Abdulrahman Bin Faisal University, Dammam, 34221, Saudi Arabia, iau.edu.sa

**Keywords:** Asia, children, prevalence, Rotavirus A, systematic review and meta-analysis

## Abstract

**Background:**

Rotavirus A remains a leading cause of acute gastroenteritis in children under 5 years of age across Asia, despite expanding vaccination programs. This study systematically synthesizes available evidence on rotavirus A detection among pediatric populations in Asian countries.

**Methods:**

We conducted a systematic review and meta‐analysis of studies reporting laboratory‐confirmed rotavirus A infection among children aged ≤ 5 years in Asia. A total of 111 studies comprising 337,019 tested children were included. Random‐effects meta‐analysis was used to estimate pooled proportions of rotavirus A positivity among tested children. Between‐study heterogeneity was assessed using the *I* ^2^ statistic.

**Results:**

The pooled proportion of rotavirus A–positive cases among tested children in Asia was 24.7% (95% CI: 22.4%–27.1%). Substantial heterogeneity was observed across studies (*I* ^2^ = 99.42%, *p* < 0.001), reflecting wide variation in study populations, diagnostic methods, and healthcare settings. Higher test positivity rates were reported in studies from Saudi Arabia (49.3%), Iran (35.2%), Malaysia (33.0%), and India (29.4%), whereas lower proportions were observed in China (13.0%) and Pakistan (18.8%). Diagnostic modality and study design significantly influenced detection rates, with ELISA‐based and cross‐sectional studies reporting higher positivity.

**Conclusion:**

Approximately one‐quarter of children tested for rotavirus A across Asia were laboratory‐positive, although estimates varied markedly across settings and thus stress the need for a routine vaccination program.

## 1. Introduction

Rotavirus A infection continues to be a persistent and widespread global health issue [1], especially among children under 5 years old, despite decades of scientific progress and the existence of vaccinations [2–4]. Rotavirus A infection, marked by acute gastroenteritis that frequently results in severe dehydration and hospitalization, disproportionately affects pediatric populations in low‐ and middle‐income countries (LMICs), particularly in the varied and densely populated areas of Asia [5, 6]. Despite a global reduction in burden attributable to extensive immunization initiatives in specific regions, rotavirus remains a predominant cause of diarrheal death and morbidity in countries with inconsistent vaccine coverage, fragmented data, and significantly varied health infrastructure [7]. The prevalence of rotavirus, which indicates the existence of antibodies reflecting prior exposure or immunity, provides essential epidemiological insights that can inform public health actions, optimize vaccine policies, and direct resource allocation [8].

The epidemiological landscape of rotavirus A infection in Asia is highly varied, influenced by intricate interactions among socioeconomic status, sanitation practices, climate variability, and healthcare accessibility [9]. Countries such as India, Bangladesh, and Indonesia have documented some of the highest hospitalization rates due to rotavirus A globally [10]. In contrast, Japan and South Korea have experienced comparatively lower incidence rates, though they encounter sporadic outbreaks [11–15].

The biology of rotavirus A infection hampers epidemiological evaluation. Rotavirus A consists of multiple genotypes and strains, with G1P [8], G2P [4], G3P [8], G9P [8], and G12P [6] being the most prevalent in Asia [3, 16, 17]. The virus demonstrates a tendency for genetic reassortment and antigenic drift, prompting valid worries over the effectiveness of current vaccinations against new strains. Genotype surveillance in Southeast Asia and South Asia has revealed significant fluctuations in predominant strains over time, accompanied by sporadic increases in atypical or zoonotic genotypes [18, 19]. These changes highlight the importance of prevalence data as an auxiliary surveillance instrument, one that illustrates the immunological environment influenced by circulating strains, previous infections, and vaccination efforts.

In nations like Pakistan, Nepal, and Myanmar, rotavirus is responsible for up to 40% of all severe diarrheal cases necessitating hospitalization in young children [20]. In such circumstances, where healthcare resources are scarce and malnutrition is widespread, rotavirus infection can rapidly escalate to a life‐threatening condition [21–23]. Although routine surveillance frequently records incidence and death statistics, prevalence investigations are essential to reveal the more nuanced, frequently obscured aspects of virus transmission and immunity, particularly in areas where clinical reporting may be unreliable or inadequately supported.

Despite numerous regional and national studies published in the last 2 decades [24–26], no comprehensive systematic analysis has consolidated prevalence estimates of rotavirus A infection for the total pediatric population of Asia.

This systematic review and meta‐analysis seek to integrate, analyze, and interpret prevalence data on rotavirus A infection among children in Asia.

## 2. Methods

### 2.1. Research Design

This investigation utilized a systematic review and meta‐analytic methodology, meticulously structured and conducted in accordance with the Preferred Reporting Items for Systematic Reviews and Meta‐Analyses (PRISMA) 2020 standards [27]. Although this review was not prospectively registered, all stages were conducted in accordance with PRISMA 2020 reporting guidelines.

All stages of the review process, including literature search, study selection, data extraction, risk‐of‐bias assessment, and quantitative synthesis, followed the PRISMA methodological guidance. A PRISMA flow diagram illustrating the study selection process and a completed PRISMA checklist and JBI criteria are provided as Supporting Files S3 and S4, respectively.

### 2.2. Criteria for Eligibility

The eligibility requirements were meticulously delineated to achieve a balance between inclusion and methodological rigor. Eligible studies were those that (i) presented original prevalence data on rotavirus infection in children aged 0–5 years; (ii) were conducted in any Asian country; (iii) employed laboratory‐confirmed serological techniques such as enzyme‐linked immunosorbent assays (ELISA), neutralization tests, or other validated antibody detection methods; and (iv) molecular techniques such as polymerase chain reaction (PCR) and PCR variants (v) were published in English up until February 2025. Conversely, studies were excluded if they were case reports, editorials, reviews, or conference abstracts, lacked prevalence data, or had a clearly defined diagnostic methodology. Studies that shared identical authorship, sampling timeframe, and geographic coverage were compared for overlapping datasets. When duplication was suspected, only the study with the largest sample size or most complete data was retained.

“Asia” was defined according to the World Health Organization (WHO) regional classification, encompassing countries within the WHO Southeast Asia Region, Western Pacific Region, and Eastern Mediterranean Region where applicable. Countries were included based on geographic location within the Asian continent and availability of eligible rotavirus A data in pediatric populations.

### 2.3. Sources of Information and Search Methodology

To guarantee comprehensive coverage, we performed a systematic search across various electronic databases, specifically PubMed, Web of Science, Embase, and Scopus, supplemented by inquiries in grey literature repositories such as ProQuest Dissertations and the WHO Global Index Medicus. Search techniques were developed utilizing a blend of Medical Subject Headings (MeSH) and free‐text keywords, carefully tailored to the syntax of each database. For example, in PubMed, our search included phrases such as (“Rotavirus” OR “Rotavirus infection” OR “Rotavirus prevalence”) AND (“Asia” OR particular Asian country names) AND (“children” OR “pediatric” OR “infant”) AND (“IgG” OR “IgM” OR “serology”) OR (PCR, PHAGE). The concluding search was finalized in February 2025. Furthermore, the references of all chosen publications and pertinent reviews were examined for any potentially overlooked studies. The complete and reproducible search strategies, including full database‐specific Boolean search strings for PubMed, Web of Science, Embase, and Scopus, are provided in Supporting File S1.

### 2.4. Data Selection and Extraction

The screening procedure employed a bifurcated review system, commencing with title and abstract assessment, followed by a comprehensive evaluation of chosen papers. The process was performed independently by seven reviewers (Reviewer 1–Reviewer 9), and inter‐reviewer agreement was measured using Cohen’s kappa coefficient, with a value exceeding 0.80, indicating considerable agreement. In instances of disagreement, a senior reviewer (Reviewer 10) facilitated the resolution process. All references were organized in the Mendeley software, and duplicates were methodically eliminated prior to the initiation of screening. The data extraction was methodically organized and conducted with precision. A standardized data extraction form was created and pilot‐tested on five randomly selected articles to verify reliability and clarity. The form recorded bibliographic information (first author, publication year), study design, nation, geographic region, sample size, serological test utilized, and prevalence estimates. Data extraction was conducted by two separate reviewers who entered the information into a precoded spreadsheet. Any discrepancies or uncertainties were resolved jointly or referred to a third reviewer for resolution.

### 2.5. Evaluation of Bias Risk

The potential for bias in individual studies was evaluated utilizing the Joanna Briggs Institute (JBI) Critical Appraisal Checklist for Prevalence/Proportion Studies [28]. This tool assesses critical methodological dimensions, including sample representativeness, sample size adequacy, outcome measurement validity, and diagnostic assay reliability. Each study was assessed for risk of bias as low, moderate, or high according to the cumulative score ranging from 0–16, as shown in Supporting file S2. The findings of this evaluation were narratively synthesized and illustrated using a risk‐of‐bias summary table.

### 2.6. Data Integration and Statistical Evaluation

To ascertain the pooled proportion of rotavirus‐specific antibodies, meta‐analytic techniques were employed utilizing the random‐effects model [29], which accommodates both intrastudy and interstudy variability. Before analysis, proportions underwent the Freeman–Tukey double arcsine transformation to stabilize variance and normalize the distribution. Pooled estimates were computed for the proportion of rotavirus, both in aggregate and categorized by location, study design, and diagnostic technique.

Heterogeneity was statistically evaluated using the *I* ^2^ statistic, with values beyond 75% indicating significant heterogeneity [30]. Subgroup analyses and meta‐regression models were performed to investigate the origins of this heterogeneity. Subgroup classifications encompassed geographic sources, assay methodologies (ELISA vs. alternatives), and study designs. Publication bias was assessed by funnel plots, complemented by Egger’s test for asymmetry [31]. All statistical analyses were performed using Comprehensive Meta‐Analysis (CMA) Version 4.0 and OpenMeta Analyst [32], both of which are extensively employed platforms for quantitative synthesis in epidemiological research. The CMA was employed to calculate pooled proportion estimates, create forest plots with exceptional precision and adaptability. OpenMeta Analyst enhanced this by facilitating comprehensive subgroup analyses, permitting the examination of variability across geographic regions, study designs, and diagnostic techniques. Statistical significance was established at *p* < 0.05, with all estimates accompanied by their respective 95% confidence ranges.

## 3. Result

### 3.1. Study Selection

The preliminary database search produced 1847 records, comprising papers obtained from PubMed, Web of Science, Embase, and Scopus, as well as entries from grey literature sources like ProQuest and WHO’s Global Index Medicus. Following the elimination of 412 duplicate entries, 1435 unique studies were retained for screening. Two reviewers independently performed title and abstract screening utilizing established inclusion and exclusion criteria. This phase resulted in the elimination of 1210 records owing to irrelevance, absence of proportion data, or ineligible demographic categories.

**FIGURE 1:**
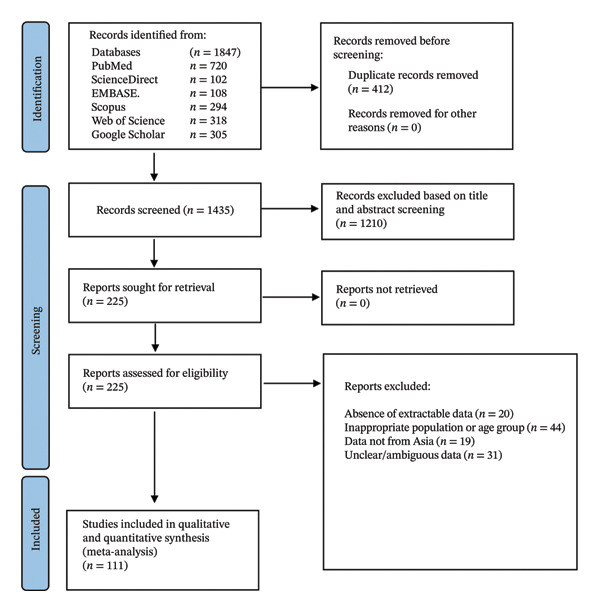
Summary of the study selection and screening process.

A total of 225 full‐text papers were obtained for comprehensive evaluation. Out of these, 156 studies were excluded following full‐text review for various reasons: lack of testing methods, absence of extractable data, inappropriate population or age group, duplicate reporting from the same dataset, and data outside Asia. In total, 111 research studies fulfilled all qualifying requirements and were incorporated into the final qualitative synthesis. All 111 studies furnished adequate quantitative data for inclusion in the meta‐analysis. Details of the screening and selection process are summarized in Figure 1.

The study selection process adhered strictly to PRISMA 2020 recommendations and is summarized in a PRISMA flow diagram (Figure 1).

### 3.2. Characteristics of the Included Studies

The studies encompass several Asian countries, providing a comprehensive picture of rotavirus proportion in children. A total of 111 studies were analyzed, involving more than 300,000 pediatric participants (Table 1). Table 1 delineates the principal characteristics of the research that examined rotavirus proportion in children across various nations. The dataset covers an extensive temporal and geographical range, including research conducted from 1990 to 2024 in Turkey, Iran, China, and various other Asian nations such as India, Pakistan, Malaysia, and Saudi Arabia.

**TABLE 1:** Characteristics of the included studies on the proportion of pediatric rotavirus A infection in Asia.

Name of author	Year of publication	Country	Total sample	Positive cases	Method of diagnosis	Study design
Akan et al. [33]	2009	Turkey	672	126	IM	Retrospective
Akpinar et al. [34]	2016	Turkey	3102	262	IM	Retrospective
Aslantaş et al. [35]	2017	Turkey	1712	289	IM	Retrospective
Atalay et al. [36]	2013	Turkey	2636	607	IM	Retrospective
Balcı et al. [37]	2010	Turkey	930	247	LA	Retrospective
Biçer et al. [38]	2011	Turkey	1543	386	IM	Retrospective
Borsa et al. [39]	2013	Turkey	944	192	IM	Retrospective
Çoban and Topal [40]	2014	Turkey	1036	422	IM	Retrospective
Doğan et al. [41]	2014	Turkey	1988	198	IM	Retrospective
Gül et al. [42]	2005	Turkey	148	38	LA	Retrospective
Gültepe et al. [43]	2012	Turkey	180	74	LA	Retrospective
Güreser et al. [44]	2017	Turkey	3189	446	IM	Retrospective
Iraz and Ceylan [45]	2013	Turkey	6749	821	IM	Retrospective
Ilktaç et al. [46]	2012	Turkey	8982	1562	IM	Retrospective
Kaşifoğlu et al. [47]	2011	Turkey	1241	247	ELISA	Retrospective
Kurugöl et al. [48]	2003	Turkey	920	366	ELISA	Retrospective
Özdemir et al. [49]	2010	Turkey	309	106	ELISA	Retrospective
Sanal [50]	2013	Turkey	5215	930	IM	Retrospective
Dağı and Fındık [51]	2014	Turkey	1793	204	IM	Retrospective
Tüzüner et al. [52]	2016	Turkey	5156	726	IM	Retrospective
Yazıcı et al. [53]	2013	Turkey	1069	244	IM	Retrospective
Alaşehir et al. [54]	2014	Turkey	920	141	IM	Retrospective
Balkan et al. [55]	2012	Turkey	340	88	IM	Retrospective
Bayraktar et al. [56]	2009	Turkey	1358	300	IM	Retrospective
Bekdaş et al. [57]	2013	Turkey	6563	987	IM	Retrospective
Berk and Kayman [58]	2012	Turkey	3445	958	IM	Retrospective
Inci et al. [59]	2009	Turkey	1258	232	IM	Retrospective
Çalgın et al. [60]	2015	Turkey	321	60	IM	Retrospective
Konca et al. [61]	2014	Turkey	3607	545	IM	Retrospective
Nazik et al. [62]	2016	Turkey	1985	147	IM	Retrospective
Tanrıverdi et al. [63]	2017	Turkey	786	104	IM	Retrospective
Ma et al. [64]	2022	China	365	67	ELISA/PCR	Sentinel surveillance
Hu et al. [65]	2021	China	418	132	ELISA/PCR	Sentinel surveillance
Cao et al. [66]	2022	China	894	216	ELISA/PCR	Sentinel surveillance
Wang et al. [67]	2022	China	306	49	ELISA/PCR	Sentinel surveillance
Huang et al. [68]	2021	China	1512	109	ELISA/PCR	Sentinel surveillance
Zhou et al. [69]	2023	China	425	94	PCR	Sentinel surveillance
Shen et al. [70]	2022	China	198,130	70,813	ELISA/PCR	Sentinel surveillance
Jiao et al. [71]	2023	China	748	82	ELISA/PCR	Sentinel surveillance
Jiang et al. [72]	2023	China	20,013	2682	ELISA/PCR	Sentinel surveillance
Zhou et al. [73]	2020	China	4409	1125	ELISA/PCR	Sentinel surveillance
Cao et al. [74]	2023	China	2560	660	PCR	Sentinel surveillance
Amini et al. [75]	1990	Iran	915	229	ELISA	Cross‐sectional
Saeb et al. [76]	1997	Iran	450	73	ELISA	Cross‐sectional
Habibi et al. [77]	2004	Iran	180	66	ELISA	Cross‐sectional
Moradi and Mokhtari [78]	2001	Iran	171	50	ELISA	Cross‐sectional
Khalili et al. [79]	2004	Iran	245	191	ELISA/PCR	Cross‐sectional
Modares et al. [80]	2005	Iran	1250	406	RNA‐PAGE, latex agglutination	Cross‐sectional
Kordidarian et al. [81]	2007	Iran	80	21	ELISA/PCR	Cross‐sectional
Kazemi et al. [82]	2006	Iran	186	57	ELISA	Cross‐sectional
Zarnani et al. [83]	2004	Iran	704	108	ELISA	Cross‐sectional
Samarbafzadeh et al. [84]	2005	Iran	137	36	ELISA	Cross‐sectional
Samarbafzadeh et al. [84]	2005	Iran	63	23	ELISA	Cross‐sectional
Kazemi et al. [85]	2007	Iran	400	126	ELISA	Cross‐sectional
Taremi et al. [86]	2005	Iran	372	95	ELISA	Cross‐sectional
Hamkar et al. [87]	2008	Iran	400	248	ELISA	Cross‐sectional
Eesteghamati et al. [88]	2009	Iran	2198	1299	ELISA	Cross‐sectional
Savadkoohi et al. [89]	2007	Iran	208	125	ELISA	Cross‐sectional
Kargar et al. [90]	2012	Iran	138	48	ELISA	Cross‐sectional
Emamghorashi et al. [91]	2015	Iran	102	69	ELISA	Cross‐sectional
Farahtaj et al. [92]	2007	Iran	374	92	ELISA	Cross‐sectional
Najafi et al. [93]	2012	Iran	138	48	ELISA/PCR	Cross‐sectional
Zaraei‐Mahmoodabadi et al. [94]	2009	Iran	193	153	ELISA	Cross‐sectional
Zaraei‐Mahmoodabadi et al. [94]	2009	Iran	67	14	ELISA	Cross‐sectional
Yahyapour et al. [95]	2008	Iran	200	100	ELISA	Cross‐sectional
Sadeghian et al. [96]	2010	Iran	156	45	ELISA	Cross‐sectional
Kargar et al. [97]	2008	Iran	260	90	ELISA	Cross‐sectional
Moradi‐Lakeh et al. [98]	2009	Iran	213	113	ELISA	Cross‐sectional
Taheri et al. [99]	2010	Iran	311	191	ELISA	Cross‐sectional
Manesh et al. [100]	2011	Iran	150	29	PAGE	Cross‐sectional
Maleki et al. [101]	2010	Iran	118	29	PAGE	Cross‐sectional
Kargar et al. [102]	2011	Iran	375	91	ELISA	Cross‐sectional
Hamkar et al. [87]	2008	Iran	353	225	ELISA	Cross‐sectional
Moradi et al. [103]	2010	Iran	411	218	PAGE	Cross‐sectional
Kargar et al. [104]	2013	Iran	141	40	ELISA	Cross‐sectional
Khoshdel et al. [105]	2014	Iran	100	30	PCR	Cross‐sectional
Rahbarimanesh and Sayari [106]	2011	Iran	700	133	PAGE	Cross‐sectional
Ghorashi et al. [107]	2011	Iran	511	285	ELISA	Cross‐sectional
Kargar et al. [108]	2010	Iran	163	75	ELISA	Cross‐sectional
Hassanzadeh and Al‐E‐Yasin [109]	2001	Iran	220	22	EM	Cross‐sectional
Moghim et al. [110]	2012	Iran	150	19	PAGE	Cross‐sectional
Jadali et al. [111]	2013	Iran	2988	1655	ELISA	Cross‐sectional
Kajbaf et al. [112]	2012	Iran	180	63	ELISA	Cross‐sectional
Motamedifar et al. [113]	2013	Iran	827	42	ELISA	Cross‐sectional
Kargar et al. [114]	2014	Iran	184	52	ELISA/PCR	Cross‐sectional
Fun et al. [115]	1991	China	419	2	ELISA	Cross‐sectional
Das et al. [116]	2013	China	6859	218	ELISA	Retrospective
Faruque et al. [117]	2004	China	1197	70	ELISA	Retrospective
Uchida et al. [118]	2006	China	260	18	ELISA	Cross‐sectional
Cheun et al. [119]	2010	China	2979	233	ELISA	Retrospective
Tatte et al. [120]	2010	China	174	15	ELISA	Cross‐sectional
Unal et al. [121]	2016	China	246	26	IC	Cross‐sectional
Hacimustafaoglu et al. [122]	2011	China	368	46	ELISA	Cross‐sectional
Wang et al. [123]	2007	China	440	85	PAGE	Cross‐sectional
Podklzin et al. [124]	2009	China	360	73	PCR	Cross‐sectional
Hung et al. [125]	2006	Malaysia	3317	1265	ELISA	Cross‐sectional
Salim et al. [126]	2017	Malaysia	158	60	ELISA	Cross‐sectional
Lee et al. [127]	2003	Malaysia	1363	333	ELISA/PCR	Cross‐sectional
Banerjee et al. [128]	2006	India	1495	176	ELISA	Retrospective
Girish Kumar et al. [129]	2020	India	25,129	7783	ELISA/PCR	Retrospective
John et al. [130]	2014	India	250	60	ELISA	Retrospective
Mathew et al. [131]	2013	India	1827	648	ELISA	Cross‐sectional
Giri et al. [132]	2019	India	5834	2069	ELISA	Cross‐sectional
Shrivastava et al. [133]	2019	India	320	98	EIA	Cross‐sectional
Sarangi et al. [134]	2015	India	265	123	EIA	Cross‐sectional
Ahmad et al. [135]	2016	Pakistan	95	9	PCR	Retrospective
Alam et al. [136]	2013	Pakistan	1306	447	EIA	Retrospective
Umair et al. [137]	2018	Pakistan	502	24	PCR	Cross‐sectional
Haque et al. [138]	2022	Pakistan	135	66	ELISA	Cross‐sectional
Tayeb et al. [139]	2011	Saudi Arabia	1007	660	ELISA	Retrospective
Meqdam and Thwiny [140]	2007	Saudi Arabia	284	94	ELISA	Cross‐sectional

Over 100 studies are documented, with a significant range in sample sizes (from 63 to over 198,000 participants), diagnostic methodologies, and research approaches. Many studies adopted retrospective or cross‐sectional designs, with sentinel surveillance being employed in recent Chinese research. China notably provided the study with the most extensive sample size [70], encompassing 198,130 children and documenting 70,813 positive cases.

Regarding diagnostic methods, ELISA was the predominant technique utilized globally, frequently supplemented or substituted by PCR, immunochromatography (IM), latex agglutination (LA), PAGE, and electron microscopy (EM). Turkey’s research was predominantly retrospective and largely dependent on immunological methods, but Iran demonstrated methodological diversity using ELISA, PAGE, PCR, and EM. Recent studies in China incorporated ELISA and PCR into national surveillance frameworks, indicating a shift toward more systematic diagnosis.

### 3.3. Risk‐of‐Bias Summary

A comprehensive risk‐of‐bias assessment was conducted for the 111 included studies using the JBI Critical Appraisal Checklist for Proportion Studies. Each study was scored on a scale of 0–16 based on methodological quality, including factors such as sample representativeness, diagnostic validity, and outcome measurement reliability. Based on these scores, studies were categorized into three levels of risk: low (13–16), moderate (10–12), and high (0–9).

**FIGURE 2:**
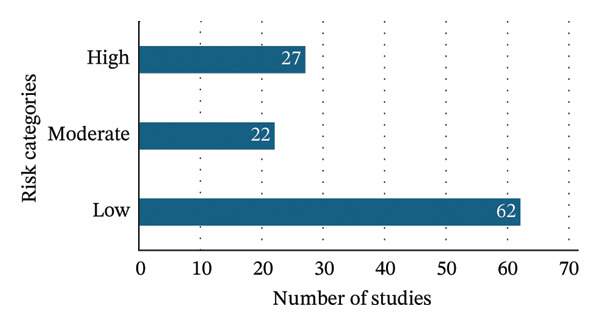
Bias risk summary chart of the included studies.

Out of the total studies assessed, 62 (55.86%) were classified as low risk, demonstrating sound methodological rigor. Twenty‐two studies (19.81%) fell into the moderate risk category, typically due to minor issues such as partial reporting or smaller sample frames. Meanwhile, 27 studies (24.32%) were identified as high risk. These high‐risk studies frequently featured small sample sizes (less than 100 participants) and reliance on outdated or less reliable diagnostic techniques. A bias risk summary diagram is presented in Figure 2, and a detailed table listing each study’s score and risk category has been provided in the Supporting file S2 section to improve transparency and satisfy reporting standards.

### 3.4. Pooled Proportion of Rotavirus in Asia

The pooled estimate derived in this meta‐analysis represents the proportion of children who tested positive for rotavirus A among those who underwent laboratory testing, rather than the population‐level proportion. Across all included studies, the pooled rotavirus A positivity proportion was 24.7% (Figure 3). Considerable heterogeneity was observed (*I* ^2^ = 99.42%, *p* < 0.001), indicating substantial variability between studies in terms of diagnostic practices, healthcare access, and study design.

FIGURE 3(a) Forest plot showing the pooled proportion of rotavirus among children in Asia. (b) Forest plot showing the pooled regional proportion of rotavirus A infection in Asia.

**Figure:**
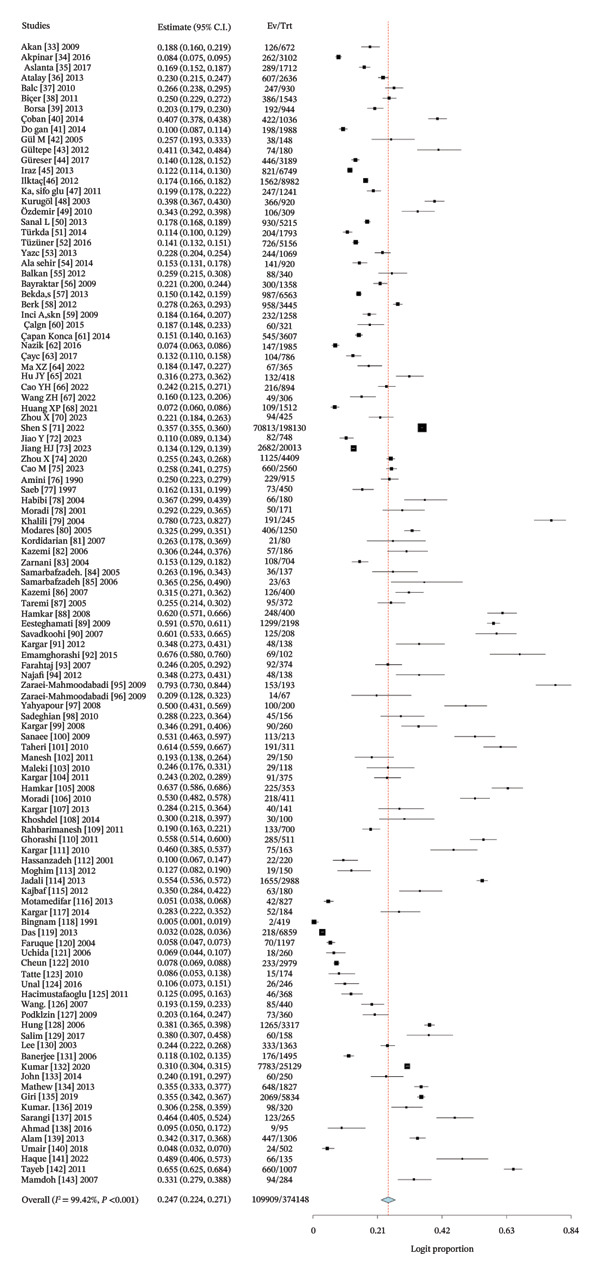
(a)

**Figure:**
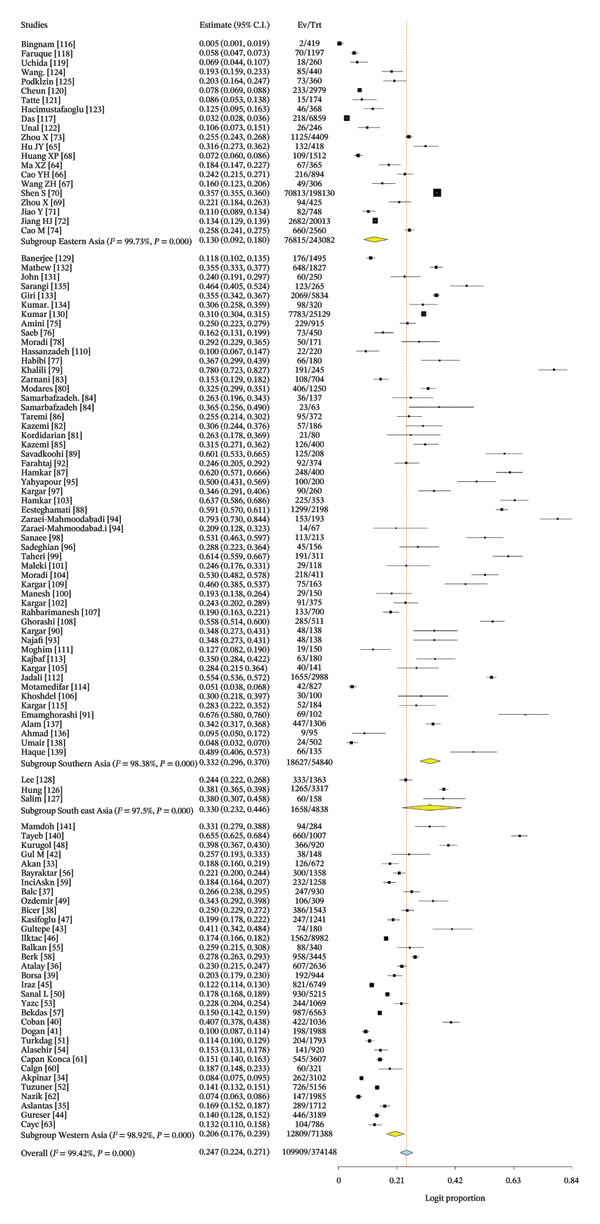
(b)

**FIGURE 4:**
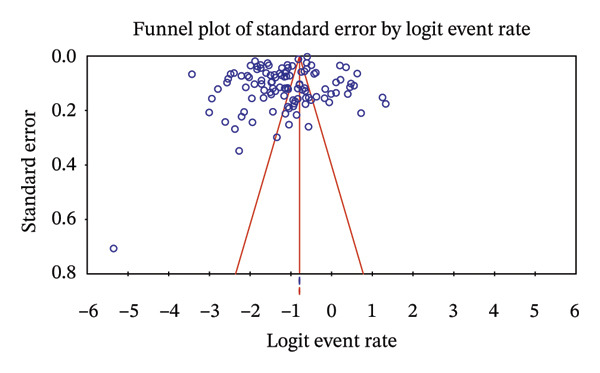
Funnel plot showing the publication bias of the proportion of rotavirus among children in Asia. Egger’s *p* = 0.00022.

The regional proportion of rotavirus A–positive cases among tested children reveals that Southeast Asia had the highest proportion (Figure 3(b)). The funnel plot and Egger’s test (*p* = 0.00022) (Figure 4) and symmetrical distribution of studies indicated no significant publication bias in the pooled proportion of rotavirus among children in Asia.

#### 3.4.1. Sensitivity Analysis of High‐Risk Studies

**FIGURE 5:**
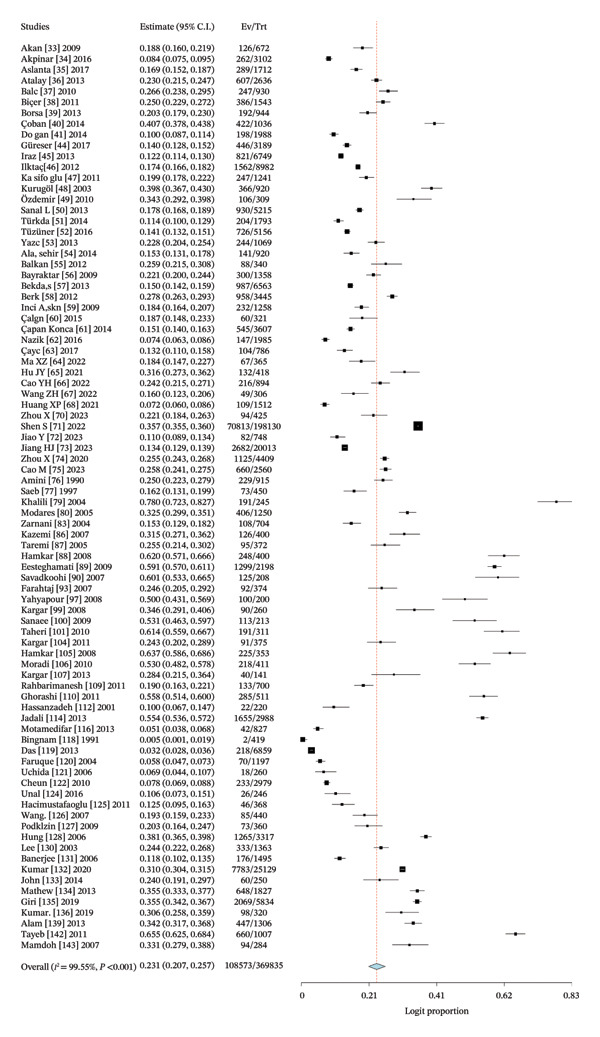
Adjusted pooled proportion of rotavirus A infection after sensitivity and bias assessment.

**FIGURE 6:**
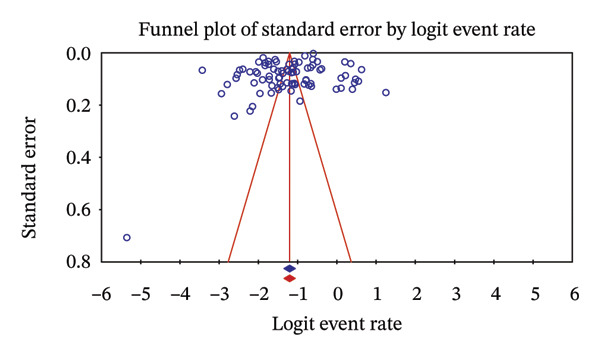
Adjusted funnel plot showing no publication bias proportion of rotavirus A infection after sensitivity and bias assessment. Egger’s *p* = 0.00009.

A sensitivity analysis was carried out by eliminating all studies found to be high risk in order to determine the sensitivity of the pooled proportion estimate to them. The sample sizes in these 27 studies were poor; this was one of the methodological issues raised by these studies. Upon their exclusion, the pooled proportion of rotavirus infection in children in Asia was slightly lower than before, 24.7% (95% CI: 22.4–27.1) to 23.1% (95% CI: 20.7–25.7) (Figure 5). Also, the adjusted pooled proportion was not biased in publication (Figure 6).

### 3.5. Subgroup Analysis

Table 2 provides a detailed subgroup analysis of rotavirus proportion in children across different Asian nations, categorized by country, detection method, and study methodology. The estimates are obtained from a binary random‐effects model utilizing logit proportions, including variability among studies.

**TABLE 2:** Subgroup meta‐analysis of rotavirus proportion among children in Asia.

Parameter	No. of studies	Estimate (%)	Confidence interval (95%)	*Q* statistics	Heterogeneity index (*I* ^2^)	DF	*p*‐value
Country							
Turkey	31	19.2	16.8–21.9	1897.385	98.42%	30	< 0.001
China	21	13.0	9.2–18.0	7364.342	99.73%	20	< 0.001
Iran	43	35.2	29.8–41.0	2210.642	98.10%	42	< 0.001
Malaysia	3	33.0	23.2–44.6	80.132	97.50%	2	< 0.001
India	7	29.4	24.7–34.7	329.611	98.18%	6	< 0.001
Pakistan	4	18.8	6.8–42.1	152.525	98.03%	3	< 0.001
Saudi Arabia	2	49.3	20.7–78.5	89.339	98.88%	1	< 0.001
Method of detection							
IM	25	17.1	14.9–19.6	1438.707	98.33%	24	< 0.001
LA	3	30.7	22.4–40.5	15.953	87.46%	2	< 0.001
ELISA	50	29.9	24.6–35.7	5955.647	99.18%	49	< 0.001
ELISA/PCR	15	24.8	20.0–30.3	4697.936	99.70%	14	< 0.001
PCR	6	16.9	11.3–24.5	96.674	94.83%	5	< 0.001
RNA‐PAGE, latex agglutination	1	32.5	29.9–35.1	NA	NA	NA	NA
PAGE	6	23.2	13.3–37.3	182.058	97.25%	5	< 0.001
EM	1	10.0	6.7–14.7	NA	NA	NA	NA
IC	1	10.6	7.3–15.1	NA	NA	NA	NA
EIA	3	36.7	29.3–44.8	17.781	88.75%	2	0.001
Type of study design							
Retrospective	40	18.6	15.7–21.9	6134.099	99.36%	39	< 0.001
Sentinel surveillance	11	19.6	13.5–27.6	4494.325	99.78%	10	< 0.001
Cross‐sectional	60	30.8	27.1–34.7	3067.475	98.08%	59	< 0.001
Time trend analysis							
1990–1996	2	1.9	0.2–18.8	12.673	92.11	1	< 0.001
1997–2003	5	11.6	6.9–19.0	95.921	95.83	4	< 0.001
2004–2010	40	25.5	22.3–29.0	7208.052	99.46	39	< 0.001
2011–2017	48	31.7	27.2–36.6	4444.645	98.94	47	< 0.001
2018–2024	16	14.8	12.4–17.6	847.084	98.23	15	< 0.001

*Note:* IM = immunochromatography, ELISA = enzyme‐linked immunosorbent assay, PAGE = polyacrylamide gel electrophoresis, IC = immunochromatography, EIA = enzyme immunoassay.

Abbreviations: EM = electron microscopy, LA = latex agglutination, NA = not applicable, PCR = polymerase chain reaction.

**FIGURE 7:**
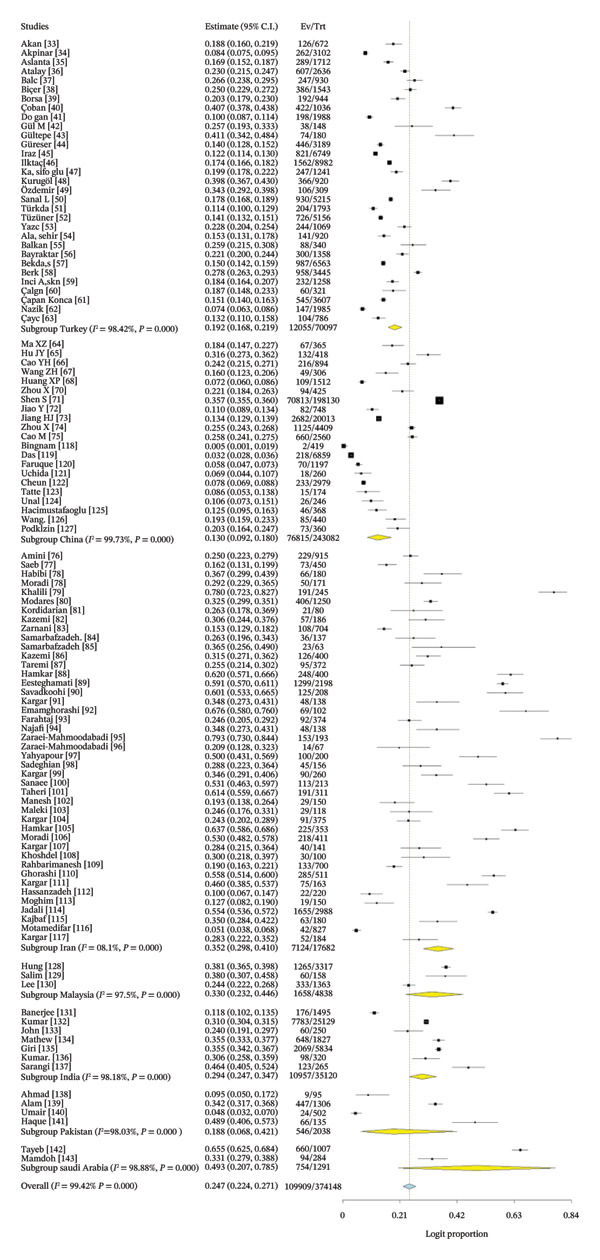
Subgroup analysis of rotavirus among children in Asia in relation to country of origin.

The incidence of rotavirus in children varies markedly by nation. The highest estimate was recorded in Saudi Arabia (49.3%) (Figure 7). The high proportion rate in Saudi Arabia needs to be interpreted with caution due to the limited sample size, and further regional research verification is needed. Iran (35.2%), Malaysia (33.0%), and India (29.4%) exhibited elevated proportion rates. Conversely, China (13.0%) and Pakistan (18.8%) exhibited lower proportion rates. Turkey, with a substantial number of studies (*n* = 31), reported a moderate frequency of 19.2% (Table 2). All country‐specific analyses exhibited exceedingly high heterogeneity (*I* ^2^ > 97%), signifying considerable variation among studies, perhaps attributable to disparities in population demographics, geographic contexts, diagnostic methodologies, or temporal frameworks.

The temporal pattern of the proportion of rotavirus A in children in Asia reveals an obvious trend in the change in its proportion over the study period. Between 1990 and 1996, this proportion was very low (1.9%), and it is unclear whether it reflects limited circulation or underdetection at the time. This was succeeded by gradual growth, which culminated to 11.6% between 1997 and 2003 and thereafter nearly doubled to 25.5% during 2004–2010. Its highest is between 2011 and 2017, when it reaches 31.7%, meaning increased transmission or enhanced detection. However, a drastic fall of 14.8% in the latest period (2018–2024) has been witnessed, and this could be attributed to the use of rotavirus vaccination and better sanitation, as well as wider public health safeguards encompassing several countries in Asia.

**FIGURE 8:**
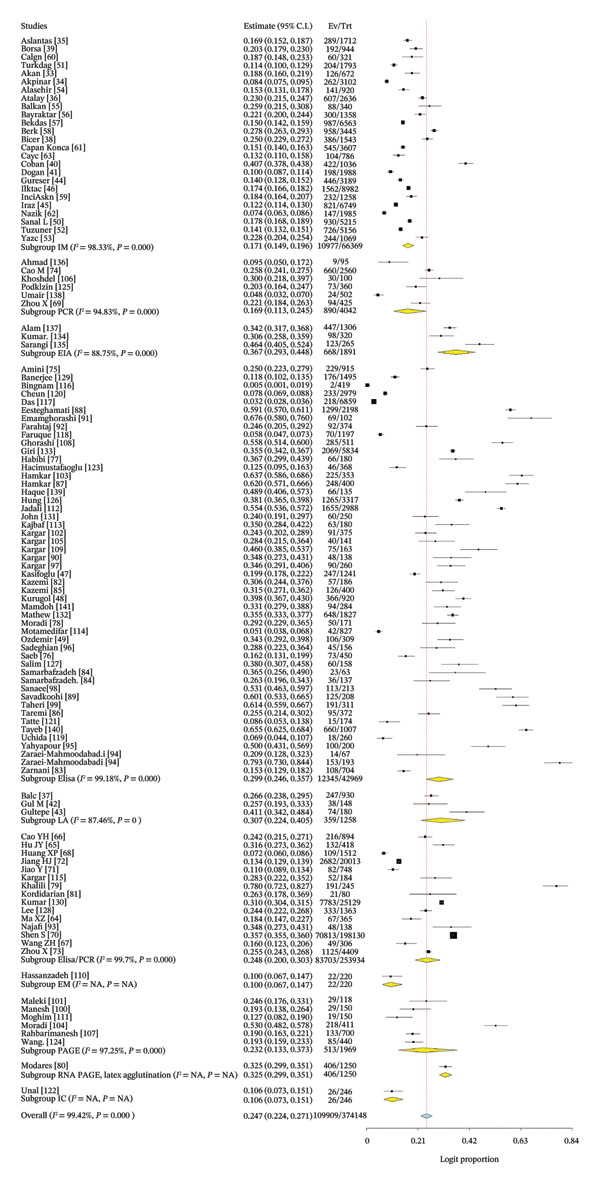
Subgroup analysis of rotavirus among children in Asia in relation to the method of detection.

Diverse diagnostic techniques produce disparate proportion estimates. EIA (36.7%), ELISA (29.9%), and LA (30.7%) had the highest proportion rates (Figure 8), presumably owing to their higher sensitivity in identifying viral antigens. Techniques such as PCR (16.9%), IM (17.1%), and EM (10.0%) exhibited reduced proportions, potentially indicating variations in diagnostic efficacy, study demographics, or reporting methodologies (Table 2).

**FIGURE 9:**
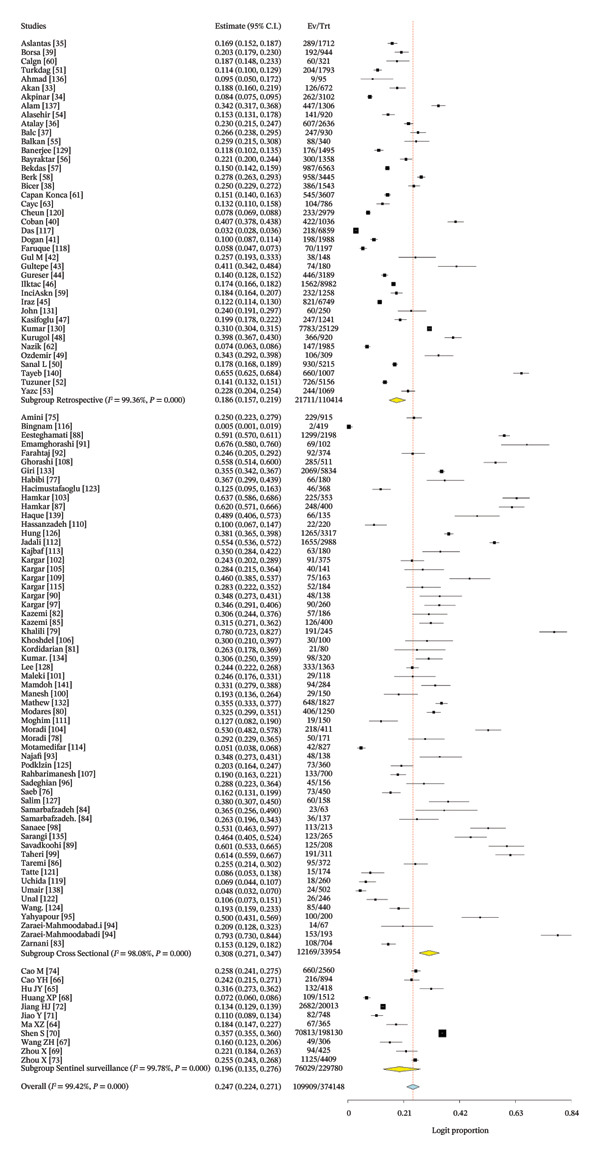
Subgroup analysis of rotavirus among children in Asia in relation to the type of study design.

The study design seemingly affects proportion estimates. Cross‐sectional studies (Table 2), often more extensive in population representation, indicated the highest frequency of rotavirus at 30.8%, followed by sentinel monitoring at 19.6% and retrospective studies at 18.6% (Figure 9). Cross‐sectional designs may identify a greater number of instances owing to expansive sampling methodologies and rigorous data acquisition.

### 3.6. Meta‐Regression Analysis

Meta‐regression was done to assess the effect of the study‐level variables on the proportion of rotavirus A–positive cases among tested children in Asia. Such variables as the country of study, the method of detection, study period, region, and temporal trends were evaluated. Assessment of variables that might be linearly related to effect size shown to exist between continuous variables was done, but variables that have a *p* value of < 0.25 were included in the multivariable model. In the last multivariate model, most of the other variables maintained their link with the RVA proportion. It is notable that the proportion rate was higher in selective countries like Saudi Arabia, Turkey, and Iran, whereas Southern Asia had a major increase in estimates as compared to Eastern Asia. Some of the years of study (2004 to 2007, 2008, 2015, 2020, and 2022) also demonstrated a significant relation to an increased proportion. A significant negative association was shown by EM only among the detection methods. There were no other significant categories of trends; however, the proportion has shown to be on the rise according to temporal trends in 20,112,017. The final multivariate meta‐regression results are presented in Table 3.

**TABLE 3:** Final meta‐regression analysis of the variables associated with rotavirus A infection in Asia.

Variable	Coefficient	*p* value	95% CI
Countries			
China	Reference		
India	1.391	< 0.001	0.662–2.120
Iran	1.687	< 0.001	1.127–2.247
Malaysia	1.495	0.002	0.558–2.432
Pakistan	0.961	0.039	0.051–1.871
Saudi Arabia	3.394	< 0.001	1.959–4.830
Turkey	1.931	< 0.001	0.964–2.899
Method of detection			
ELISA	Reference		
EIA	0.605	0.196	−0.311–1.522
ELISA/PCR	0.013	0.962	−0.534–0.561
EM	−1.681	0.018	−3.067–(−)0.294
IC	0.069	0.927	−1.388–1.525
IM	−0.758	0.063	−1.556–0.040
LA	0.006	0.991	−1.068–1.081
PAGE	−0.384	0.200	−0.972–0.203
PCR	−0.180	0.606	−0.862–0.502
RNA‐PAGE, latex agglutination	−0.215	0.749	−1.535–1.105
Trends			
1990–1996	Reference		
1997–2003	1.230	0.116	−0.302–2.762
2004–2010	1.446	0.067	−0.102–2.994
2011–2017	1.819	0.041	0.071–3.568
2018–2024	1.354	0.148	−0.482–3.190
Regions			
Eastern Asia	Reference		
Southeast Asia	1.074	0.081	−0.134–2.282
Southern Asia	0.986	0.008	0.261–1.712
Western Asia	0.603	0.163	−0.244–1.449
Study period			
1991	Reference		
2004	2.561	0.020	0.401–4.721
2007	2.341	0.048	0.016–4.666
2008	2.922	0.015	0.575–5.268
2015	2.637	0.031	0.244–5.030
2020	2.464	0.048	0.027–4.901
2021	2.228	0.083	−0.29.2–4.749
2022	2.647	0.032	0.232–5.062
2023	2.326	0.063	−0.125–4.777

### 3.7. Spatial Distribution of Rotavirus in Asia

**FIGURE 10:**
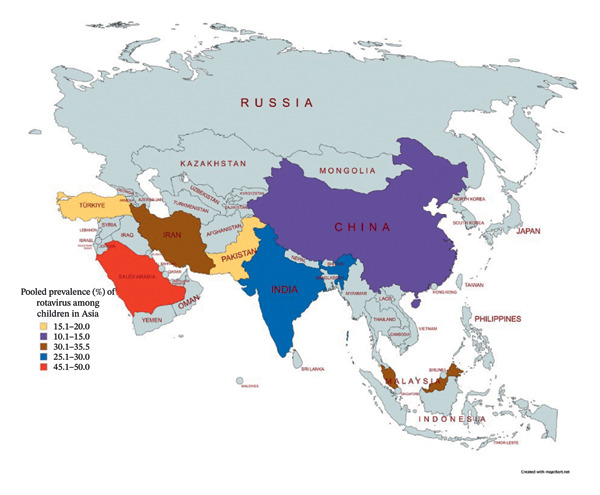
Spatial distribution of rotavirus among children in Asia.

The regional distribution of rotavirus proportion among children in Asia shows significant variation, with Saudi Arabia reporting the highest proportion, followed by Iran, Turkey, and China, the lowest as represented in Figure 10.

In East Asia, China exhibits the lowest frequency at 13.0%, as illustrated in Figure 8, possibly due to effective national surveillance systems, widespread immunization initiatives in recent years, and prompt diagnosis and care. Conversely, adjacent South Asian nations like India (29.4%) and Pakistan (18.8%) demonstrate comparatively elevated rates, possibly because of differences in vaccination accessibility, healthcare provision, and sanitation standards. The comparatively lower percentage in Pakistan relative to India may be attributed to underreporting or limited‐scale investigations.

Southeast Asia, exemplified by Malaysia (33.0%), exhibits a notably high frequency, potentially reflecting deficiencies in vaccination uptake or monitoring sensitivity despite its status as a comparatively affluent nation in the region (Figure 8).

In the Middle East and Central Asia, Iran demonstrates a significant frequency of 35.2%, while Saudi Arabia records the greatest proportion overall at 49.3%. The data indicate regional endemicity, potentially intensified by environmental conditions that promote viral persistence and seasonal transmission peaks. The notably high proportion in Saudi Arabia may indicate variations in diagnostic sensitivity or biases in hospital‐based sampling. Turkey, with 19.2%, exemplifies a transitional pattern situated between Europe and Asia, with a proportion rate lower than that of Iran and its Middle Eastern counterparts, yet higher than China’s.

## 4. Discussion

Rotavirus is a significant contributor to acute gastroenteritis in children under five, especially in Asia [11]. The aggregated proportion of rotavirus infection in children within the region was determined to be 24.7%, underscoring its considerable impact. This discovery corresponds with global epidemiological patterns documented by others [6, 141], which recognized rotavirus as a predominant contributor to diarrheal mortality in young children, with Asia representing a significant share of this burden. Likewise, research conducted by others reported an elevated proportion rate in South and Southeast Asia before and during the early stages of vaccine implementation [11, 142].

The 21.1% proportion indicates that around one in five children with diarrhea in Asia are infected with rotavirus, highlighting its ongoing transmission despite the presence of vaccinations in various nations. Variations in vaccine coverage, healthcare accessibility, and the execution of sanitation and hygiene measures may exacerbate this persistent illness load [8]. Some Asian countries have made substantial advancements in incorporating the rotavirus vaccine into their national immunization programs, while others remain deficient, resulting in inconsistent protection throughout the region [143].

Significant publication bias indicates that studies with a greater proportion rate are more likely to be disseminated. This bias may obscure the actual disease burden by under‐representing research with minimal or no findings. The pooled estimate reported in this study should not be interpreted as a direct measure of population‐level proportion. Rather, it reflects the proportion of laboratory‐confirmed rotavirus A cases among children who were tested, most often in healthcare settings. Hospital‐based sampling and heterogeneous testing indications likely resulted in preferential inclusion of more severe gastroenteritis cases, thereby inflating positivity estimates relative to the community‐based proportion. This distinction is critical when comparing results across countries and regions with differing healthcare infrastructures.

This subgroup meta‐analysis reveals the significant burden and intricate epidemiology of rotavirus infection in children throughout Asia. The disparity in frequency among countries, diagnostic techniques, and research methodologies highlights the complex factors influencing rotavirus transmission and detection, indicating a necessity for enhanced standardization and public health measures throughout the region. The markedly elevated incidence in Saudi Arabia and Iran relative to Turkey and China aligns with existing evidence, indicating that areas with diminished rotavirus vaccine coverage and inadequate sanitary facilities typically exhibit higher infection rates [9].

The restricted availability of routine rotavirus vaccination in Iran during the initial years of the study may account for the heightened proportion. Conversely, China has achieved significant advancements in the implementation of rotavirus vaccines via both private and governmental health sectors, potentially elucidating the relatively lower incidence rate [24]. Turkey, located between Asia and Europe, exhibits intermediate vaccine deployment and health infrastructure, potentially influencing its moderate proportion estimate [144].

Intercountry variations are further exacerbated by disparities in diagnostic procedures, which are essential for disease diagnosis and reporting. ELISA, utilized in almost half of the papers examined, is acknowledged for its convenience and moderate sensitivity; nonetheless, research indicates it may be less effective than PCR in identifying low viral loads [145, 146]. The pooled proportion of 22.8% among ELISA users aligns with other research conducted in Asian contexts utilizing a similar methodology. Conversely, PCR‐based assays, recognized for their elevated sensitivity and specificity, indicated a marginally lower pooled frequency of 20.4%. However, their use may have been skewed toward more affluent urban centers or research facilities. The highest incidence was observed in investigations employing LA and hybrid ELISA/PCR methodologies. These findings corroborate prior conclusions by Bonica et al., who underscore that the choice of diagnostic assays substantially influences observed rotavirus epidemiology, especially in resource‐constrained environments [147].

A notable observation is the impact of study design on the proportion outcomes. Cross‐sectional studies demonstrated the greatest pooled proportion, presumably representing snapshots of rotavirus incidence during peak transmission periods. These studies are typically more temporally and geographically focused, frequently concentrating on high‐incidence environments such as hospitals or outbreaks [148–150]. Conversely, retrospective studies, exhibiting a moderate pooled proportion, may under‐represent the actual frequency owing to insufficient records or misclassification of cases. Sentinel surveillance is intended to track illness trends over time. Although structured and systematic, sentinel data frequently reflect selected populations and may overlook community‐based cases, as indicated in worldwide assessments by the WHO Rotavirus Surveillance Network [7, 151].

The considerable heterogeneity (*I* ^2^ > 98%) across all groupings signifies substantial variability both between and within nations and methodologies. This degree of heterogeneity aligns with findings from other extensive rotavirus meta‐analyses conducted in underdeveloped nations [152].

A critical result of this approach is the necessity for standardized diagnostic and methodological frameworks. Considering the noted inconsistencies in proportion based on diagnostic procedures, standardizing detection techniques could enhance data comparability and facilitate more dependable public health interventions. Moreover, there is an urgent necessity for systematic rotavirus immunization initiatives throughout Asia. The WHO advocates for the incorporation of rotavirus vaccines across all national immunization programs; nevertheless, implementation has been inconsistent throughout the continent [151].

Among these methods, ELISA and EIA are common types of antigen detection methods with reported sensitivity varying between 85 and 90%, especially in acute pediatric rotavirus infection in the framework of stool samples being taken during peak viral shedding. The ease and inexpensiveness of their operations, together with the capability to identify antigens of viruses when they remain lingering past the acute stage of the disease, make the use of these tests widespread in resource‐constrained settings [7, 152].

Although sensitivity and specificity data were not reported in most included studies, variations in diagnostic performance likely contributed to differences in proportion estimates. Furthermore, the timing of sample collection may influence detection rates, as earlier or later sampling could affect pathogen detectability.

However, the possibilities of detection of low concentrations of viral RNA due to the abilities of detecting subclinical infection or low viral load make PCR assays, RT‐PCR, and nested PCR analytically more sensitive, with sensitivity often higher than 95% [3, 4]. Nonetheless, in PCR‐based studies, it is not uncommon to find a low proportion of rotavirus when compared with ELISA/EIA‐based studies, even with the high sensitivity value. It can be explained by the fact that ELISA and EIA are viral antigen detectors, and they can be found in the stool longer after the active infection period has passed, hence covering both active infections and previous infections. Instead of that, PCR needs intact viral RNA, which is more prone to degradation and possibly subject to timing, handling, or storage, and can be underdetected when samples are taken outside the optimal detection timeline [153, 154].

Considered together, ELISA and PCR are more sensitive than simpler, more rapid tests, including IM and LA (an approximate sensitivity range of 60%–80%). There are more false‐negative tests, especially when working with samples containing low viral load or late infections, and thus, there is a possibility of underestimating the rotavirus proportion [155]. The other method used in a few studies is EM, which is more specific, but much less sensitive and tedious, and thus not feasible to be used in routine diagnosis [156]. As is evident based on this meta‐analysis, the decision regarding diagnostic approach has implications for the estimation of the proportion. The proportion rates were also higher in studies that were based on ELISA or EIA diagnoses but lower in studies that used PCR. Such diagnostic variability underlines the requirements of standardization of diagnostic procedures in epidemiology monitoring to allow the comparability of the studies across jurisdictions.

Data from nations implementing universal rotavirus vaccination, like India and China, indicate substantial reductions in hospitalization and mortality associated with diarrheal illnesses [25, 156]. A crucial insight is the significance of surveillance infrastructure in comprehending and managing the dissemination of rotavirus. Sentinel monitoring programs must be augmented and fortified to guarantee they accurately reflect the wider population demographics and regional diversity.

The geographic distribution of rotavirus proportion among children in Asia, as demonstrated by country‐level statistics, indicates significant geographical variances that may indicate variations in public health infrastructure, vaccine coverage, and socioeconomic development.

Significant disparities in rotavirus proportion were noted among countries. Saudi Arabia exhibited the highest occurrence, although this figure is derived from merely two studies, indicating a possible overestimation or restricted representativeness. Comparable high proportion rates in Iran, Malaysia, and India indicate that rotavirus is a significant contributor to diarrheal illnesses in these areas. These findings correspond with another report, where they documented similar proportion rates in LMICs in Asia and the Middle East, influenced by disparities in vaccine coverage, sanitation practices, and healthcare accessibility [146].

It is important to acknowledge that a substantial proportion of the included studies originated from Iran, which may have influenced the pooled proportion estimates. Iran has a long‐standing research focus on pediatric gastroenteritis and relatively extensive rotavirus surveillance compared with several other Asian countries, resulting in a higher volume of published proportion studies. This overrepresentation may partly amplify country‐specific estimates in regional analyses. However, subgroup analyses, sensitivity analyses excluding high‐risk studies, and multivariable meta‐regression demonstrated that elevated proportion estimates in Iran remained significant even after adjustment for study design, diagnostic method, and study period. These findings suggest that the observed proportion reflects a genuine epidemiological burden rather than a methodological artifact alone.

Pooled estimates indicate that Iran had a high rotavirus proportion. The variation in proportion seems to correspond with regional disparities in rotavirus control initiatives. Eastern Asia, exemplified by China, has achieved notable advancements in enhancing immunization coverage and upgrading sanitation facilities. Although rotavirus vaccines are not now included in China’s official immunization program, the adoption of vaccines in the private sector and enhancements in urban healthcare may have led to a comparatively low proportion [26].

The proportion rate of 35.2% in West Asia, namely, in Iran, indicates substantial deficiencies in vaccination accessibility and utilization. Rotavirus vaccinations have not been universally adopted in Iran, and several studies incorporated in the meta‐analysis were conducted before or during the initial phases of vaccine implementation [157–160]. Moreover, varied diagnostic capacities across regions, socioeconomic inequities, and inadequate public health infrastructure in rural areas may have intensified the illness burden.

Turkey, situated at the confluence of Asia and Europe, embodies a transitional zone both geographically and epidemiologically. The intermediate proportion rate may indicate incomplete immunization coverage and disparities in healthcare access between urban and rural populations. Despite Turkey’s introduction of rotavirus vaccinations into the commercial sector, they have not been incorporated into the national immunization schedule, thus constraining their efficacy in reducing disease at the population level [159].

### 4.1. Strengths and Limitations of the Study

This study offers the most thorough synthesis to date of rotavirus prevalence in children across Asia, amalgamating data from more than 100 studies spanning various nations. The enormous sample size and stringent methods, encompassing subgroup and sensitivity analyses, provide substantial, region‐specific insights that can guide targeted vaccination policies and public health measures. The considerable heterogeneity across the included studies, stemming from variations in diagnostic methodologies, research designs, and demographic characteristics, may restrict the generalizability of the aggregated results and create potential bias in cross‐national comparisons. Most of the included studies were hospital‐based and relied on testing children who presented with gastroenteritis, limiting generalizability to the broader pediatric population. Second, heterogeneity was extremely high across all analyses, driven by differences in diagnostic methods, healthcare access, surveillance systems, and study design. Third, variations in national testing strategies and vaccine uptake preclude direct inference of true population‐level disease burden from pooled estimates. The presence of small sample subgroups in countries like Malaysia and Saudi Arabia results in insufficient stability and reliability of data. Because the majority of included studies were hospital‐based, the prevalence estimates may be biased toward more severe cases. Consequently, asymptomatic or mild infections in the community may be underrepresented, and the true community prevalence could be higher than reported.

The unequal distribution of studies across countries, particularly the overrepresentation of data from Iran and Turkey, may have introduced regional weighting effects despite the use of random‐effects models. Another variation in diagnostic methods and healthcare access across countries may have contributed to heterogeneity. Some countries in Central and Southeast Asia remain underrepresented due to limited published data. It is important to note that cross‐country and regional comparisons should be interpreted cautiously. Testing strategies, healthcare‐seeking behavior, diagnostic availability, and indications for stool testing vary widely across Asian countries. As a result, observed differences in rotavirus A positivity rates may reflect healthcare system characteristics and surveillance intensity rather than true differences in underlying population burden.

This review was limited to studies published in English. As a result, important evidence reported in other languages, such as Chinese, Japanese, Korean, and Southeast Asian languages, may have been omitted, potentially introducing language bias.

## 5. Conclusions

This systematic review and meta‐analysis highlight the enduring and geographically diverse burden of rotavirus infection in children across Asia, notwithstanding ongoing vaccination initiatives. The aggregated prevalence estimates of 24.7% indicate significant ongoing transmission, especially in areas with restricted vaccination availability and insufficient public health infrastructure. The significant variability among countries, diagnostic methods, and study designs underscores essential disparities in surveillance capabilities and healthcare provision throughout the continent.

## Author Contributions

Ali A. Rabaan and Abdulsalam Alawfi conceived the study and provided overall supervision. Bello E. Kizito and Amer Alshengeti contributed to the systematic search strategy and literature screening. Amal H. Alfaraj, Wadha A. Alfouzan, Haya Altawalah, and Dalal Meshal Bashah conducted data extraction and quality assessment. Alya Aali Almatrafi, Bandar Alwan Albaradi, Mohammed Garout, and Heba A. Alsaffar performed the statistical analyses and interpretation. Mona A. Al‐Zaher, Noor M. Al Sheef, Esraa Z. Al‐Nass, and Leia Kamal Mohammed AlKhathlan contributed to data visualization and figure preparation. Hayam A. Alrasheed, Nawal A. AlKaabi, Zainab H. Almansour, and Huseyin Tombuloglu reviewed the final draft for intellectual content and methodological accuracy.

## Funding

No funding was available for this study.

## Disclosure

All authors contributed to drafting the manuscript, critically revised it for important intellectual content, and approved the final version for submission.

## Ethics Statement

The authors have nothing to report.

## Consent

The authors have nothing to report.

## Conflicts of Interest

The authors declare no conflicts of interest.

## Supporting Information

S1: Search strategy. S2: JBI quality checklist for the included data. S3: PRISMA 2020 Checklist. S4: JBI Checklist for Prevalence data.

## Supporting information


**Supporting Information** Additional supporting information can be found online in the Supporting Information section.

## Data Availability

The data that support the findings of this study are available from the corresponding author upon reasonable request.
