# Supplementary material for: Pediatric Rotavirus A Infection Among 337,019 Participants Within Asia: A Pan‐Asian Systematic Review and Meta‐Analysis
Source: J Trop Med. 2026 Apr 29;2026:6627946. doi: 10.1155/jotm/6627946 (PMC13126254; doi:10.1155/jotm/6627946)
Supplement: Supplementary file 1 — Supporting Information Additional supporting information can be found online in the Supporting Information section. [file JOTM-2026-6627946-s001.zip › SUPP FILE/S2 QUALITY OF INCLUDED STUDIES BY JBI CRITICAL APPRAISAL CHECKLIST FOR STUDIES REPORTING PREVALENCE DATA.docx]

**QUALITY OF INCLUDED STUDIES BY JBI CRITICAL APPRAISAL CHECKLIST FOR STUDIES REPORTING PREVALENCE DATA**

| S/N | Name of authors [reference] and year of publication | | JBI checklist* | | | | | | | | | **Total JBI score (0 -16)** | **Risk category** |
| --- | --- | --- | --- | --- | --- | --- | --- | --- | --- | --- | --- | --- | --- |
|  |  |  | 1 | 2 | 3 | 4 | 5 | 6 | 7 | 8 | 9 |  |  |
| 1 | Akan [33] | 2009 | Yes | No | Yes | Yes | Yes | Yes | Yes | Yes | Yes | 16 | Low risk |
| 2 | Akpinar [34] | 2016 | Yes | No | Yes | Yes | Yes | Yes | Yes | Yes | Yes | 16 | Low risk |
| 3 | Aslantaş [35] | 2017 | Yes | No | Yes | Yes | Yes | Yes | Yes | Yes | Yes | 16 | Low risk |
| 4 | Atalay [36] | 2013 | Yes | No | Yes | Yes | Yes | Yes | Yes | Yes | Yes | 16 | Low risk |
| 5 | Balcı [37] | 2010 | Yes | No | Yes | Yes | Yes | Yes | Yes | Yes | Yes | 16 | Low risk |
| 6 | Biçer [38] | 2011 | Yes | No | Yes | Yes | Yes | Yes | Yes | Yes | Yes | 16 | Low risk |
| 7 | Borsa [39] | 2013 | Yes | No | Yes | Yes | Yes | Yes | Yes | Yes | Yes | 16 | Low risk |
| 8 | Çoban [40] | 2014 | Yes | No | Yes | Yes | Yes | Yes | Yes | Yes | Yes | 16 | Low risk |
| 9 | Do ˘gan [41] | 2014 | Yes | No | Yes | Yes | Yes | Yes | Yes | Yes | Yes | 16 | Low risk |
| 10 | Gül M [42] | 2005 | Yes | No | Yes | No | Yes | No | Yes | No | No | 8 | High risk |
| 11 | Gültepe [43] | 2012 | Yes | No | Yes | No | Yes | No | Yes | No | No | 8 | High risk |
| 12 | Güreser [44] | 2017 | Yes | No | Yes | Yes | Yes | Yes | Yes | Yes | Yes | 16 | Low risk |
| 13 | Iraz [45] | 2013 | Yes | No | Yes | Yes | Yes | Yes | Yes | Yes | Yes | 16 | Low risk |
| 14 | Ilktaç[46] | 2012 | Yes | No | Yes | Yes | Yes | Yes | Yes | Yes | Yes | 16 | Low risk |
| 15 | Ka¸sifo ˘glu [47] | 2011 | Yes | No | Yes | Yes | Yes | Yes | Yes | Yes | Yes | 16 | Low risk |
| 16 | Kurugöl [48] | 2003 | Yes | No | Yes | Yes | Yes | Yes | Yes | Yes | Yes | 16 | Low risk |
| 17 | Özdemir [49] | 2010 | Yes | No | Yes | Yes | Yes | No | Yes | No | Yes | 12 | Moderate risk |
| 18 | Sanal L [50] | 2013 | Yes | No | Yes | Yes | Yes | Yes | Yes | Yes | Yes | 16 | Low risk |
| 19 | Türkdağı [51] | 2014 | Yes | No | Yes | Yes | Yes | Yes | Yes | Yes | Yes | 16 | Low risk |
| 20 | Tüzüner [52] | 2016 | Yes | No | Yes | Yes | Yes | Yes | Yes | Yes | Yes | 16 | Low risk |
| 21 | Yazıcı [53] | 2013 | Yes | No | Yes | Yes | Yes | Yes | Yes | Yes | Yes | 16 | Low risk |
| 22 | Ala¸sehir [54] | 2014 | Yes | No | Yes | Yes | Yes | Yes | Yes | Yes | Yes | 16 | Low risk |
| 23 | Balkan [55] | 2012 | Yes | No | Yes | Yes | Yes | No | Yes | No | Yes | 12 | Moderate risk |
| 24 | Bayraktar [56] | 2009 | Yes | No | Yes | Yes | Yes | Yes | Yes | Yes | Yes | 16 | Low risk |
| 25 | Bekda¸s [57] | 2013 | Yes | No | Yes | Yes | Yes | Yes | Yes | Yes | Yes | 16 | Low risk |
| 26 | Berk [58] | 2012 | Yes | No | Yes | Yes | Yes | Yes | Yes | Yes | Yes | 16 | Low risk |
| 27 | Inci A¸skın [59] | 2009 | Yes | Yes | Yes | Yes | Yes | Yes | Yes | Yes | Yes | 18 | Low risk |
| 28 | Çalgın [60] | 2015 | Yes | No | Yes | Yes | Yes | No | Yes | No | Yes | 12 | Moderate risk |
| 29 | Çapan Konca [61] | 2014 | Yes | No | Yes | Yes | Yes | Yes | Yes | Yes | Yes | 16 | Low risk |
| 30 | Nazik [62] | 2016 | Yes | No | Yes | Yes | Yes | Yes | Yes | Yes | Yes | 16 | Low risk |
| 31 | Çaycı [63] | 2017 | Yes | No | Yes | Yes | Yes | Yes | Yes | Yes | Yes | 16 | Low risk |
| 32 | Ma XZ [64] | 2022 | Yes | No | Yes | Yes | Yes | No | Yes | No | Yes | 12 | Moderate risk |
| 33 | Hu JY [65] | 2021 | Yes | No | Yes | Yes | Yes | Yes | Yes | Yes | Yes | 16 | Low risk |
| 34 | Cao YH [66] | 2022 | Yes | No | Yes | Yes | Yes | Yes | Yes | Yes | Yes | 16 | Low risk |
| 35 | Wang ZH [67] | 2022 | Yes | No | Yes | Yes | Yes | No | Yes | No | Yes | 12 | Moderate risk |
| 36 | Huang XP [68] | 2021 | Yes | No | Yes | Yes | Yes | Yes | Yes | Yes | Yes | 16 | Low risk |
| 37 | Zhou X [70] | 2023 | Yes | Yes | Yes | Yes | Yes | Yes | Yes | Yes | Yes | 18 | Low risk |
| 38 | Shen S [71] | 2022 | Yes | No | Yes | Yes | Yes | Yes | Yes | Yes | Yes | 16 | Low risk |
| 39 | Jiao Y [72] | 2023 | Yes | Yes | Yes | Yes | Yes | Yes | Yes | Yes | Yes | 18 | Low risk |
| 40 | Jiang HJ [73] | 2023 | Yes | No | Yes | Yes | Yes | Yes | Yes | Yes | Yes | 16 | Low risk |
| 41 | Zhou X [74] | 2020 | Yes | Yes | Yes | Yes | Yes | Yes | Yes | Yes | Yes | 18 | Low risk |
| 42 | Cao M [75] | 2023 | Yes | No | Yes | Yes | Yes | Yes | Yes | Yes | Yes | 16 | Low risk |
| 43 | Amini [76] | 1990 | Yes | No | Yes | Yes | Yes | Yes | Yes | Yes | Yes | 16 | Low risk |
| 44 | Saeb [77] | 1997 | Yes | No | Yes | Yes | Yes | Yes | Yes | Yes | Yes | 16 | Low risk |
| 45 | Habibi [78] | 2004 | Yes | No | Yes | No | Yes | No | Yes | No | No | 8 | High risk |
| 46 | Moradi [78] | 2001 | Yes | No | Yes | No | Yes | No | Yes | No | No | 8 | High risk |
| 47 | Khalili [79] | 2004 | Yes | No | Yes | Yes | Yes | No | Yes | No | Yes | 12 | Moderate risk |
| 48 | Modares [80] | 2005 | Yes | No | Yes | Yes | Yes | Yes | Yes | Yes | Yes | 16 | Low risk |
| 49 | Kordidarian [81] | 2007 | Yes | No | Yes | No | Yes | No | Yes | No | No | 8 | High risk |
| 50 | Kazemi [82] | 2006 | Yes | No | Yes | No | Yes | No | Yes | No | No | 8 | High risk |
| 51 | Zarnani [83] | 2004 | Yes | No | Yes | Yes | Yes | Yes | Yes | Yes | Yes | 16 | Low risk |
| 52 | Samarbafzadeh. [84] | 2005 | Yes | No | Yes | No | Yes | No | Yes | No | No | 8 | High risk |
| 53 | Samarbafzadeh [85] | 2006 | Yes | No | Yes | No | Yes | No | Yes | No | No | 8 | High risk |
| 54 | Kazemi [86] | 2007 | Yes | Yes | Yes | Yes | Yes | Yes | Yes | Yes | Yes | 18 | Low risk |
| 55 | Taremi [87] | 2005 | Yes | Yes | Yes | Yes | Yes | Yes | Yes | Yes | Yes | 18 | Low risk |
| 56 | Hamkar [88] | 2008 | Yes | No | Yes | Yes | Yes | Yes | Yes | Yes | Yes | 16 | Low risk |
| 57 | Eesteghamati [89] | 2009 | Yes | No | Yes | Yes | Yes | Yes | Yes | Yes | Yes | 16 | Low risk |
| 58 | Savadkoohi [90] | 2007 | Yes | No | Yes | Yes | Yes | No | Yes | No | Yes | 12 | Moderate risk |
| 59 | Kargar [91] | 2012 | Yes | No | Yes | No | Yes | No | Yes | No | No | 8 | High risk |
| 60 | Emamghorashi [92] | 2015 | Yes | No | Yes | No | Yes | No | Yes | No | No | 8 | High risk |
| 61 | Farahtaj [93] | 2007 | Yes | No | Yes | Yes | Yes | No | Yes | No | Yes | 12 | Moderate risk |
| 62 | Najafi [94] | 2012 | Yes | No | Yes | No | Yes | No | Yes | No | No | 8 | High risk |
| 63 | Zaraei-Mahmoodabadi [95] | 2009 | Yes | No | Yes | No | Yes | No | Yes | No | No | 8 | High risk |
| 64 | Zaraei-Mahmoodabadi [96] | 2009 | Yes | No | Yes | No | Yes | No | Yes | No | No | 8 | High risk |
| 65 | Yahyapour [97] | 2008 | Yes | No | Yes | Yes | Yes | Yes | Yes | Yes | Yes | 16 | Moderate risk |
| 66 | Sadeghian [98] | 2010 | Yes | No | Yes | No | Yes | No | Yes | No | No | 8 | High risk |
| 67 | Kargar [99] | 2008 | Yes | No | Yes | Yes | Yes | No | Yes | No | Yes | 12 | Moderate risk |
| 68 | Sanaee[100] | 2009 | Yes | No | Yes | Yes | Yes | No | Yes | No | Yes | 12 | Moderate risk |
| 69 | Taheri [101] | 2010 | Yes | No | Yes | Yes | Yes | No | Yes | No | Yes | 12 | Moderate risk |
| 70 | Manesh [102] | 2011 | Yes | No | Yes | No | Yes | No | Yes | No | No | 8 | High risk |
| 71 | Maleki [103] | 2010 | Yes | No | Yes | No | Yes | No | Yes | No | No | 8 | High risk |
| 72 | Kargar [104] | 2011 | Yes | No | Yes | Yes | Yes | No | Yes | No | Yes | 12 | Moderate risk |
| 73 | Hamkar [105] | 2008 | Yes | No | Yes | Yes | Yes | No | Yes | No | Yes | 12 | Moderate risk |
| 74 | Moradi [106] | 2010 | Yes | No | Yes | Yes | Yes | Yes | Yes | Yes | Yes | 16 | Low risk |
| 75 | Kargar [107] | 2013 | Yes | No | Yes | Yes | Yes | Yes | Yes | Yes | Yes | 16 | Low risk |
| 76 | Khoshdel [108] | 2014 | Yes | No | Yes | No | Yes | No | Yes | No | No | 8 | High risk |
| 77 | Rahbarimanesh [109] | 2011 | Yes | No | Yes | Yes | Yes | Yes | Yes | Yes | Yes | 16 | Low risk |
| 78 | Ghorashi [110] | 2011 | Yes | No | Yes | Yes | Yes | Yes | Yes | Yes | Yes | 16 | Low risk |
| 79 | Kargar [111] | 2010 | Yes | No | Yes | No | Yes | No | Yes | No | No | 8 | High risk |
| 80 | Hassanzadeh [112] | 2001 | Yes | No | Yes | Yes | Yes | No | Yes | No | Yes | 12 | Moderate risk |
| 81 | Moghim [113] | 2012 | Yes | No | Yes | No | Yes | No | Yes | No | No | 8 | High risk |
| 82 | Jadali [114] | 2013 | Yes | No | Yes | Yes | Yes | Yes | Yes | Yes | Yes | 16 | Low risk |
| 83 | Kajbaf [115] | 2012 | Yes | No | Yes | No | Yes | No | Yes | No | No | 8 | High risk |
| 84 | Motamedifar [116] | 2013 | Yes | No | Yes | Yes | Yes | Yes | Yes | Yes | Yes | 16 | Low risk |
| 85 | Kargar [117] | 2014 | Yes | No | Yes | No | Yes | No | Yes | No | No | 8 | High risk |
| 86 | Bingnam [118] | 1991 | Yes | No | Yes | Yes | Yes | Yes | Yes | Yes | Yes | 16 | Low risk |
| 87 | Das [119] | 2013 | Yes | No | Yes | Yes | Yes | Yes | Yes | Yes | Yes | 16 | Low risk |
| 88 | Faruque [120] | 2004 | Yes | No | Yes | Yes | Yes | Yes | Yes | Yes | Yes | 16 | Low risk |
| 89 | Uchida [121] | 2006 | Yes | No | Yes | Yes | Yes | No | Yes | No | Yes | 12 | Moderate risk |
| 90 | Cheun [122] | 2010 | Yes | No | Yes | Yes | Yes | Yes | Yes | Yes | Yes | 16 | Low risk |
| 91 | Tatte [123] | 2010 | Yes | No | Yes | Yes | Yes | Yes | Yes | Yes | Yes | 16 | High risk |
| 92 | Unal [124] | 2016 | Yes | No | Yes | No | Yes | No | Yes | No | No | 8 | Moderate risk |
| 93 | Hacimustafaoglu [125] | 2011 | Yes | No | Yes | Yes | Yes | No | Yes | No | Yes | 12 | Moderate risk |
| 94 | Wang. [126] | 2007 | Yes | No | Yes | Yes | Yes | No | Yes | No | Yes | 12 | Moderate risk |
| 95 | Podklzin [127] | 2009 | Yes | No | Yes | Yes | Yes | No | Yes | No | Yes | 12 | Moderate risk |
| 96 | Hung [128] | 2006 | Yes | No | Yes | Yes | Yes | Yes | Yes | Yes | Yes | 16 | Low risk |
| 97 | Salim [129] | 2017 | Yes | No | Yes | No | Yes | No | Yes | No | No | 8 | High risk |
| 98 | Lee [130] | 2003 | Yes | No | Yes | Yes | Yes | Yes | Yes | Yes | Yes | 16 | Low risk |
| 99 | Banerjee [131] | 2006 | Yes | No | Yes | Yes | Yes | Yes | Yes | Yes | Yes | 16 | Low risk |
| 100 | Kumar [132] | 2020 | Yes | No | Yes | Yes | Yes | Yes | Yes | Yes | Yes | 16 | Low risk |
| 101 | John [133] | 2014 | Yes | No | Yes | Yes | Yes | No | Yes | No | Yes | 12 | Moderate risk |
| 102 | Mathew [134] | 2013 | Yes | No | Yes | Yes | Yes | Yes | Yes | Yes | Yes | 16 | Low risk |
| 103 | Giri [135] | 2019 | Yes | No | Yes | Yes | Yes | Yes | Yes | Yes | Yes | 16 | Low risk |
| 104 | Kumar. [136] | 2019 | Yes | No | Yes | Yes | Yes | No | Yes | No | Yes | 12 | Moderate risk |
| 105 | Sarangi [137] | 2015 | Yes | No | Yes | No | Yes | No | Yes | No | No | 8 | High risk |
| 106 | Ahmad [138] | 2016 | Yes | No | Yes | No | Yes | No | Yes | No | No | 8 | High risk |
| 107 | Alam [139] | 2013 | Yes | No | Yes | Yes | Yes | Yes | Yes | Yes | Yes | 16 | Low risk |
| 108 | Umair [140] | 2018 | Yes | No | Yes | No | Yes | No | Yes | No | No | 8 | High risk |
| 109 | Haque [141] | 2022 | Yes | No | Yes | No | Yes | No | Yes | No | No | 8 | High risk |
| 110 | Tayeb [142] | 2011 | Yes | No | Yes | Yes | Yes | Yes | Yes | Yes | Yes | 16 | Low risk |
| 111 | Mamdoh [143] | 2007 | Yes | No | Yes | Yes | Yes | No | Yes | No | Yes | 12 | Moderate risk |

**JBI CHECKLIST* 1.** Appropriate sampling frame to address target population, **2.** Appropriate sampling way of study participants, **3.** Adequate sample size, **4.** Detail description of study participants and settings, **5.** Data analysis with sufficient coverage of identified sample, **6.** Use of valid methods to identify the condition, **7.** Standard, reliable way of measurement of condition for all participants, **8.** Availability of appropriate statistical analysis, **9.** Adequate response rate and management of low response rate.

**Scores are coded as Yes=2 and No=0.**
