# Supplementary material for: Pediatric Rotavirus A Infection Among 337,019 Participants Within Asia: A Pan‐Asian Systematic Review and Meta‐Analysis
Source: J Trop Med. 2026 Apr 29;2026:6627946. doi: 10.1155/jotm/6627946 (PMC13126254; doi:10.1155/jotm/6627946)
Supplement: Supplementary file 1 — Supporting Information Additional supporting information can be found online in the Supporting Information section. [file JOTM-2026-6627946-s001.zip › SUPP FILE/S1 Search_Strategy_Rotavirus.docx]

# Search Strategy for the prevalence of Rotavirus among children in Asia

## PubMed

("Rotavirus"[MeSH Terms] OR "rotavirus infection"[MeSH Terms] OR rotavirus[tiab] OR "rotavirus infection"[tiab] OR "rotavirus seroprevalence"[tiab])
AND
("Asia"[MeSH Terms] OR Asia[tiab] OR "Asian countries"[tiab] OR "China"[tiab] OR "India"[tiab] OR "Iran"[tiab] OR "Turkey"[tiab] OR "Japan"[tiab] OR "South Korea"[tiab] OR "Pakistan"[tiab])
AND
("Child"[MeSH Terms] OR child*[tiab] OR pediatric*[tiab] OR infant*[tiab] OR adolescent*[tiab])
AND
("Seroprevalence"[MeSH Terms] OR seroprevalence[tiab] OR "IgG"[tiab] OR "IgA"[tiab] OR "antibody"[tiab] OR "serology"[tiab])

## Scopus

(TITLE-ABS-KEY(rotavirus OR "rotavirus infection" OR "rotavirus seroprevalence"))
AND
(TITLE-ABS-KEY(child* OR pediatric* OR infant* OR adolescent*))
AND
(TITLE-ABS-KEY(Asia OR "Asian countries" OR China OR India OR Iran OR Turkey OR Japan OR "South Korea" OR Pakistan))
AND
(TITLE-ABS-KEY(seroprevalence OR IgG OR IgA OR antibody OR serology))

## ScienceDirect

("rotavirus" OR "rotavirus infection" OR "rotavirus seroprevalence")
AND
("child" OR "children" OR "pediatric" OR "infant" OR "adolescent")
AND
("Asia" OR "Asian countries" OR "China" OR "India" OR "Iran" OR "Turkey" OR "Japan" OR "South Korea" OR "Pakistan")
AND
("seroprevalence" OR "IgG" OR "IgA" OR "antibody" OR "serology")

## Embase

('rotavirus'/exp OR 'rotavirus infection'/exp OR rotavirus:ti,ab OR 'rotavirus infection':ti,ab OR 'rotavirus seroprevalence':ti,ab)
AND
('child'/exp OR child*:ti,ab OR pediatric*:ti,ab OR infant*:ti,ab OR adolescent*:ti,ab)
AND
('Asia'/exp OR Asia:ti,ab OR 'Asian countries':ti,ab OR China:ti,ab OR India:ti,ab OR Iran:ti,ab OR Turkey:ti,ab OR Japan:ti,ab OR 'South Korea':ti,ab OR Pakistan:ti,ab)
AND
('seroprevalence'/exp OR seroprevalence:ti,ab OR IgG:ti,ab OR IgA:ti,ab OR antibody:ti,ab OR serology:ti,ab)

## Google Scholar

"rotavirus" AND "seroprevalence" AND ("children" OR "pediatric" OR "infants") AND ("Asia" OR "China" OR "India" OR "Iran" OR "Turkey" OR "Japan" OR "South Korea") AND ("IgG" OR "IgA" OR "serology")

## Web of Science

TS=(rotavirus OR "rotavirus infection" OR "rotavirus seroprevalence")
AND
TS=(child* OR pediatric* OR infant* OR adolescent*)
AND
TS=(Asia OR "Asian countries" OR China OR India OR Iran OR Turkey OR Japan OR "South Korea" OR Pakistan)
AND
TS=(seroprevalence OR IgG OR IgA OR antibody OR serology)
